# Supplementary material for: Rocks of different mineralogy show different temperature characteristics: implications for biodiversity on rocky seashores
Source: PeerJ. 2021 Jan 26;9:e10712. doi: 10.7717/peerj.10712 (PMC7845524; doi:10.7717/peerj.10712)
Supplement: Appendix S1 [file peerj-09-10712-s001.docx]

*The following appendix accompanies the article*

**Rocks of different mineralogy show different temperature characteristics: implications for biodiversity on rocky seashores**

**Nathan Janetzki, Kirsten Benkendorff & Peter G. Fairweather**

*Corresponding author: [nathan.janetzki@outlook.com](mailto:nathan.janetzki@outlook.com)

*Thermal imagery and false-colour scales*

Thermal imagery captures the amount of infrared energy (i.e. heat) emitted by objects in an image. When a false-colour scale is applied to each image, differences in the amount of infrared energy emitted by objects are represented by the different colours that appear (Figure 2). This false-colour scale, which is scaled separately for a range on each image, consists of eight distinct colours from black (coldest) to white (hottest). Black, purple, blue and green colours represent objects emitting less infrared energy and are thus cooler in temperature, while yellow, orange, red and white represent objects emitting more infrared energy and are thus hotter in temperature (Figure 2).

*Minimum temperatures*

Minima behaved similarly to maxima over four hours exposure with the same trends identified for weather condition (hotter minima on sunny days) and exposure time (increased with time exposed and peaked at four hours). After four hours, the hottest minimum recorded was 50.9 °C (sunny day, air temperature = 39 °C) for the upper surface of a grey siltstone boulder while the coolest minimum was 7.0 °C (sunny day, air temperature = 12 °C) for the lower surface of a fossiliferous sandstone boulder. Over four hours, increases in minimum surface temperature of >20 °C were recorded for some rocks on several days, with the greatest increases recorded for upper surfaces on sunny days. When rock types were ranked from hottest to coolest for mean minimum temperature after four hours, the same consistent ranking identified for maxima was observed (Table A2). The rock type rank order for maxima and minima was significantly correlated for both upper (Spearman’s Rho = 1.00, *p*-value < 0.01) and lower (Spearman’s Rho = 0.94, *p*-value < 0.01) surfaces. Therefore, the lower surfaces of white limestone had the coolest temperatures of the six rock types investigated. These coolest locations on white limestone potentially offer intertidal biota the greatest refuge from extreme heat on sunny days with the hottest air temperatures. The rank order of rock types according to changes in minima over four hours conformed to the consistent rankings identified elsewhere, with the two siltstones having the largest overall increases and white limestone and quartzite generally having the smallest (Table A2).

Table A1: Frequencies of occurrence (%) for the three patterns of temperature difference identified on boulder upper and lower surfaces, for each rock type, for a subset of the cloudy and sunny days sampled.

|  | Rock type: | | | | Purple siltstone | Grey siltstone | Quartzite | Fossiliferous sandstone | Orange limestone | White limestone | Total |
| --- | --- | --- | --- | --- | --- | --- | --- | --- | --- | --- | --- |
| Weather | Date | Maximum air temperature (°C) | Surface | Temperature pattern |  |  |  |  |  |  |  |
| Cloudy | 09/09/2015 | 15 | Upper | Mosaic | 0 | 0 | 0 | 0 | 0 | 0 | 0 |
|  |  |  |  | Gradient | 0 | 0 | 0 | 0 | 0 | 0 | 0 |
|  |  |  |  | Limited heterogeneity | 100 | 100 | 100 | 100 | 100 | 100 | 100 |
|  |  |  | Lower | Mosaic | 0 | 0 | 0 | 0 | 0 | 0 | 0 |
|  |  |  |  | Gradient | 0 | 0 | 0 | 0 | 0 | 0 | 0 |
|  |  |  |  | Limited heterogeneity | 100 | 100 | 100 | 100 | 100 | 100 | 100 |
|  | 25/11/2015 | 30 | Upper | Mosaic | 0 | 0 | 0 | 0 | 0 | 0 | 0 |
|  |  |  |  | Gradient | 0 | 0 | 0 | 0 | 0 | 0 | 0 |
|  |  |  |  | Limited heterogeneity | 100 | 100 | 100 | 100 | 100 | 100 | 100 |
|  |  |  | Lower | Mosaic | 0 | 0 | 0 | 0 | 0 | 0 | 0 |
|  |  |  |  | Gradient | 0 | 0 | 0 | 0 | 0 | 0 | 0 |
|  |  |  |  | Limited heterogeneity | 100 | 100 | 100 | 100 | 100 | 100 | 100 |
|  | 19/12/2015 | 38 | Upper | Mosaic | 0 | 0 | 0 | 0 | 0 | 0 | 0 |
|  |  |  |  | Gradient | 0 | 0 | 0 | 0 | 0 | 0 | 0 |
|  |  |  |  | Limited heterogeneity | 100 | 100 | 100 | 100 | 100 | 100 | 100 |
|  |  |  | Lower | Mosaic | 0 | 0 | 0 | 0 | 0 | 0 | 0 |
|  |  |  |  | Gradient | 0 | 0 | 0 | 16.7 | 0 | 16.7 | 5.6 |
|  |  |  |  | Limited heterogeneity | 100 | 100 | 100 | 83.3 | 100 | 83.3 | 94.4 |
| Sunny | 16/07/2016 | 12 | Upper | Mosaic | 0 | 0 | 83.3 | 0 | 0 | 0 | 13.9 |
|  |  |  |  | Gradient | 100 | 100 | 16.7 | 100 | 100 | 100 | 86.1 |
|  |  |  |  | Limited heterogeneity | 0 | 0 | 0 | 0 | 0 | 0 | 0 |
|  |  |  | Lower | Mosaic | 0 | 0 | 83.3 | 0 | 0 | 0 | 13.9 |
|  | Rock type: | | | | Purple siltstone | Grey siltstone | Quartzite | Fossiliferous sandstone | Orange limestone | White limestone | Total |
| Weather | Date | Maximum air temperature (°C) | Surface | Temperature pattern |  |  |  |  |  |  |  |
|  |  |  |  | Gradient | 83.3 | 100 | 16.7 | 100 | 83.3 | 100 | 80.5 |
|  |  |  |  | Limited heterogeneity | 16.7 | 0 | 0 | 0 | 16.7 | 0 | 5.6 |
|  | 07/02/2016 | 29 | Upper | Mosaic | 0 | 0 | 83.3 | 33.3 | 0 | 16.7 | 22.2 |
|  |  |  |  | Gradient | 100 | 100 | 16.7 | 66.7 | 100 | 66.6 | 75 |
|  |  |  |  | Limited heterogeneity | 0 | 0 | 0 | 0 | 0 | 16.7 | 2.8 |
|  |  |  | Lower | Mosaic | 0 | 0 | 83.3 | 33.3 | 33.3 | 0 | 25 |
|  |  |  |  | Gradient | 100 | 100 | 16.7 | 66.7 | 66.7 | 100 | 75 |
|  |  |  |  | Limited heterogeneity | 0 | 0 | 0 | 0 | 0 | 0 | 0 |
|  | 18/11/2015 | 40 | Upper | Mosaic | 0 | 0 | 16.7 | 0 | 0 | 0 | 2.8 |
|  |  |  |  | Gradient | 33.3 | 50 | 0 | 16.7 | 0 | 16.7 | 19.4 |
|  |  |  |  | Limited heterogeneity | 66.7 | 50 | 83.3 | 83.3 | 100 | 83.3 | 77.8 |
|  |  |  | Lower | Mosaic | 0 | 0 | 33.3 | 0 | 0 | 0 | 5.6 |
|  |  |  |  | Gradient | 33.3 | 16.7 | 0 | 66.7 | 33.3 | 50 | 33.3 |
|  |  |  |  | Limited heterogeneity | 66.7 | 83.3 | 66.6 | 33.3 | 66.7 | 50 | 61.1 |

Table A2: The daily rank order of rock types from largest to smallest (6 = largest, 1 = smallest) minimum temperature after four hours and change in minimum temperature over four hours for upper and lower surfaces. The rank sum (sum of daily ranks) was used to assign an overall rank to each rock type from largest to smallest (6 = largest value, 1 = smallest).

|  | Surface | Upper | | | | | | | | Lower | | | | | | | |
| --- | --- | --- | --- | --- | --- | --- | --- | --- | --- | --- | --- | --- | --- | --- | --- | --- | --- |
| Measure | Daily rank  Rock type | 6 | 5 | 4 | 3 | 2 | 1 | Rank sum | **Overall rock rank** | 6 | 5 | 4 | 3 | 2 | 1 | Rank sum | **Overall rock rank** |
| Minimum temperature after four hours | Purple siltstone | 14 | 3 | 0 | 0 | 0 | 0 | 99 | **6** | 14 | 3 | 0 | 0 | 0 | 0 | 99 | **6** |
|  | Grey siltstone | 3 | 14 | 0 | 0 | 0 | 0 | 88 | **5** | 3 | 14 | 0 | 0 | 0 | 0 | 88 | **5** |
|  | Fossiliferous sandstone | 0 | 0 | 13 | 4 | 0 | 0 | 64 | **4** | 0 | 0 | 16 | 1 | 0 | 0 | 67 | **4** |
|  | Orange limestone | 0 | 0 | 4 | 13 | 0 | 0 | 55 | **3** | 0 | 0 | 1 | 12 | 4 | 0 | 48 | **3** |
|  | White limestone | 0 | 0 | 0 | 0 | 14 | 3 | 31 | **2** | 0 | 0 | 0 | 0 | 4 | 13 | 21 | **1** |
|  | Quartzite | 0 | 0 | 0 | 0 | 3 | 14 | 20 | **1** | 0 | 0 | 0 | 4 | 9 | 4 | 34 | **2** |
|  |  |  |  |  |  |  |  |  |  |  |  |  |  |  |  |  |  |
| Change in minimum temperature over four hours | Purple siltstone | 13 | 3 | 0 | 1 | 0 | 0 | 96 | **6** | 12 | 5 | 0 | 0 | 0 | 0 | 97 | **6** |
|  | Grey siltstone | 3 | 9 | 4 | 0 | 0 | 1 | 80 | **5** | 5 | 12 | 0 | 0 | 0 | 0 | 90 | **5** |
|  | Fossiliferous sandstone | 0 | 4 | 10 | 2 | 1 | 0 | 68 | **4** | 0 | 0 | 8 | 4 | 5 | 0 | 54 | **4** |
|  | Orange limestone | 0 | 0 | 2 | 12 | 2 | 1 | 49 | **3** | 0 | 0 | 3 | 6 | 5 | 3 | 43 | **2** |
|  | White limestone | 1 | 0 | 0 | 1 | 9 | 6 | 33 | **2** | 0 | 0 | 1 | 1 | 4 | 11 | 26 | **1** |
|  | Quartzite | 0 | 1 | 1 | 1 | 5 | 9 | 31 | **1** | 0 | 0 | 5 | 6 | 3 | 3 | 47 | **3** |

Table A3: Major mineral content (%, mean ± SE) of six seashore rock types. The dominant major mineral in each rock type is bolded.

| Major mineral | Chemical formula | Grey siltstone | Purple siltstone | Quartzite | Fossiliferous sandstone | White limestone | Orange limestone |
| --- | --- | --- | --- | --- | --- | --- | --- |
| Silicon dioxide | SiO₂ | **55.4 ± 6.6** | **71.2 ± 3.8** | **96.6 ± 0.1** | **44.0 ± 4.7** | **71.6 ± 3.1** | 11.3 ± 2.3 |
| Calcium oxide | CaO | 12.0 ± 4.2 | 1.9 ± 0.2 | 0 ± 0 | 24.5 ± 0.6 | 12.3 ± 1.8 | **46.9 ± 1.4** |
| Aluminium oxide | Al₂O₃ | 8.8 ± 0.6 | 8.2 ± 1.6 | 0.7 ± 0.1 | 1.1 ± 0.1 | 1.2 ± 0.3 | 0.7 ± 0.1 |
| Iron oxide | Fe₂O₃ | 4.1 ± 0.6 | 7.3 ± 1.7 | 0.3 ± 0.1 | 3.1 ± 0.1 | 0.9 ± 0.1 | 2.1 ± 0.2 |
| Sodium oxide | Na₂O | 1.3 ± 0.4 | 2.9 ± 1.1 | 0.1 ± 0 | 0.2 ± 0.1 | 0.6 ± 0.1 | 0.3 ± 0.1 |
| Titanium dioxide | TiO₂ | 0.6 ± 0.1 | 2.0 ± 0.6 | 0.1 ± 0 | 0.1 ± 0 | 0.1 ± 0 | 0.1 ± 0 |
| Magnesium oxide | MgO | 3.3 ± 0.1 | 1.6 ± 0.1 | 0.1 ± 0 | 3.0 ± 1.9 | 0.6 ± 0.1 | 1.0 ± 0.1 |
| Potassium oxide | K₂O | 2.1 ± 0.1 | 0.9 ± 0.2 | 0 ± 0 | 0.4 ± 0 | 0.5 ± 0.1 | 0.3 ± 0.1 |
| Manganese oxide | MnO₂ | 0.1 ± 0 | 0.1 ± 0 | 0 ± 0 | 0 ± 0 | 0 ± 0 | 0 ± 0 |
| Phosphorus pentoxide | P₄O₁₀ | 0.1 ± 0 | 0.2 ± 0.1 | 0.1 ± 0 | 0.1 ± 0 | 0.1 ± 0 | 0.1 ± 0 |
| Sulfur trioxide | SO₃ | 0 ± 0 | 0.1 ± 0.1 | 0 ± 0 | 0.1 ± 0 | 0.2 ± 0 | 0.1 ± 0 |

| Element | Grey siltstone | Purple siltstone | Quartzite | Fossiliferous sandstone | White limestone | Orange limestone |
| --- | --- | --- | --- | --- | --- | --- |
| Chlorine | 192 ± 105 | 1 ± 0 | 116 ± 6 | **1526 ± 887** | **4884 ± 882** | **3687 ± 1041** |
| Barium | 288 ± 32 | **1798 ± 1358** | 185 ± 30 | 62 ± 8 | 42 ± 3 | 54 ± 2 |
| Manganese | **622 ± 77** | 852 ± 133 | 7 ± 3 | 133 ± 13 | 30 ± 11 | 255 ± 80 |
| Zirconium | 198 ± 13 | 235 ± 39 | 23 ± 1 | 126 ± 24 | 76 ± 11 | 52 ± 6 |
| Strontium | 145 ± 46 | 78 ± 22 | 25 ± 5 | 265 ± 125 | 157 ± 16 | 320 ± 64 |
| Cobalt | 66 ± 30 | 155 ± 66 | **286 ± 131** | 24 ± 4 | 46 ± 22 | 11 ± 6 |
| Copper | 35 ± 17 | 115 ± 57 | 1 ± 0 | 1 ± 0 | 1 ± 0 | 1 ± 0 |
| Vanadium | 84 ± 19 | 83 ± 8 | 3 ± 2 | 104 ± 6 | 16 ± 2 | 20 ± 2 |
| Rubidium | 79 ± 5 | 37 ± 8 | 2 ± 1 | 13 ± 1 | 17 ± 3 | 12 ± 2 |
| Chromium | 52 ± 6 | 28 ± 5 | 16 ± 10 | 45 ± 6 | 18 ± 3 | 12 ± 2 |
| Zinc | 68 ± 9 | 37 ± 15 | 1 ± 0 | 7 ± 1 | 6 ± 1 | 6 ± 1 |
| Arsenic | 2 ± 1 | 7 ± 1 | 3 ± 1 | 55 ± 3 | 6 ± 1 | 20 ± 1 |
| Bismuth | 2 ± 1 | 3 ± 1 | 2 ± 1 | 2 ± 1 | 2 ± 1 | 1 ± 0 |
| Bromine | 1 ± 0 | 1 ± 0 | 1 ± 0 | 4 ± 2 | 11 ± 2 | 7 ± 1 |
| Cadmium | 2 ± 1 | 3 ± 1 | 2 ± 1 | 2 ± 1 | 2 ± 1 | 2 ± 1 |
| Cerium | 39 ± 5 | 32 ± 7 | 1 ± 0 | 16 ± 8 | 1 ± 0 | 7 ± 6 |
| Caesium | 7 ± 6 | 1 ± 0 | 1 ± 0 | 8 ± 4 | 1 ± 0 | 11 ± 6 |
| Gallium | 12 ± 1 | 9 ± 1 | 2 ± 0 | 1 ± 0 | 3 ± 1 | 3 ± 0 |
| Germanium | 2 ± 0 | 3 ± 0 | 2 ± 0 | 1 ± 0 | 1 ± 0 | 1 ± 0 |
| Iodine | 1 ± 0 | 1 ± 0 | 1 ± 0 | 23 ± 6 | 1 ± 0 | 14 ± 4 |
| Lanthanum | 17 ± 9 | 6 ± 5 | 7 ± 6 | 1 ± 0 | 1 ± 0 | 1 ± 0 |
| Niobium | 9 ± 1 | 18 ± 4 | 1 ± 0 | 3 ± 0 | 2 ± 1 | 2 ± 0 |
| Neodymium | 20 ± 3 | 15 ± 8 | 8 ± 4 | 4 ± 3 | 4 ± 3 | 7 ± 6 |
| Nickel | 9 ± 3 | 7 ± 6 | 1 ± 0 | 14 ± 13 | 1 ± 0 | 1 ± 0 |
| Lead | 7 ± 1 | 9 ± 2 | 4 ± 2 | 3 ± 1 | 4 ± 0 | 3 ± 0 |
| Scandium | 3 ± 2 | 5 ± 2 | 1 ± 0 | 1 ± 0 | 1 ± 0 | 1 ± 0 |
| Samarium | 1 ± 0 | 5 ± 4 | 1 ± 0 | 1 ± 0 | 1 ± 0 | 1 ± 0 |
| Thorium | 18 ± 1 | 16 ± 2 | 10 ± 0 | 13 ± 0 | 10 ± 0 | 11 ± 1 |
| Thallium | 8 ± 1 | 8 ± 1 | 8 ± 2 | 7 ± 1 | 6 ± 1 | 7 ± 1 |
| Uranium | 6 ± 2 | 3 ± 0 | 2 ± 1 | 5 ± 1 | 5 ± 0 | 9 ± 1 |
| Ytterbium | 1 ± 0 | 7 ± 3 | 12 ± 1 | 1 ± 0 | 1 ± 0 | 1 ± 0 |
| Yttrium | 21 ± 2 | 25 ± 5 | 14 ± 1 | 4 ± 0 | 4 ± 0 | 3 ± 0 |

Table A4: Trace element content (parts per million, mean ± SE) of six seashore rock types. Silver, molybdenum, hafnium, antimony, selenium, tin, tantalum and tellurium were also detected but were too small to be quantitatively measured. The dominant trace element in each rock type is bolded.


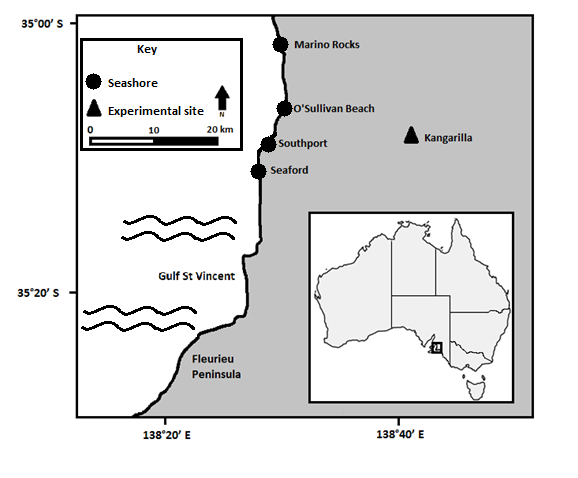


Figure A1: Four seashores where boulders were collected from South Australia’s Fleurieu Peninsula, and the inland location, Kangarilla, where the common-garden experiment was completed. Purple or grey siltstone was collected from Marino Rocks, quartzite from O’Sullivan Beach, white or orange fossiliferous limestone from Southport and fossiliferous sandstone from Seaford. Inset map shows the location of the study region within Australia.


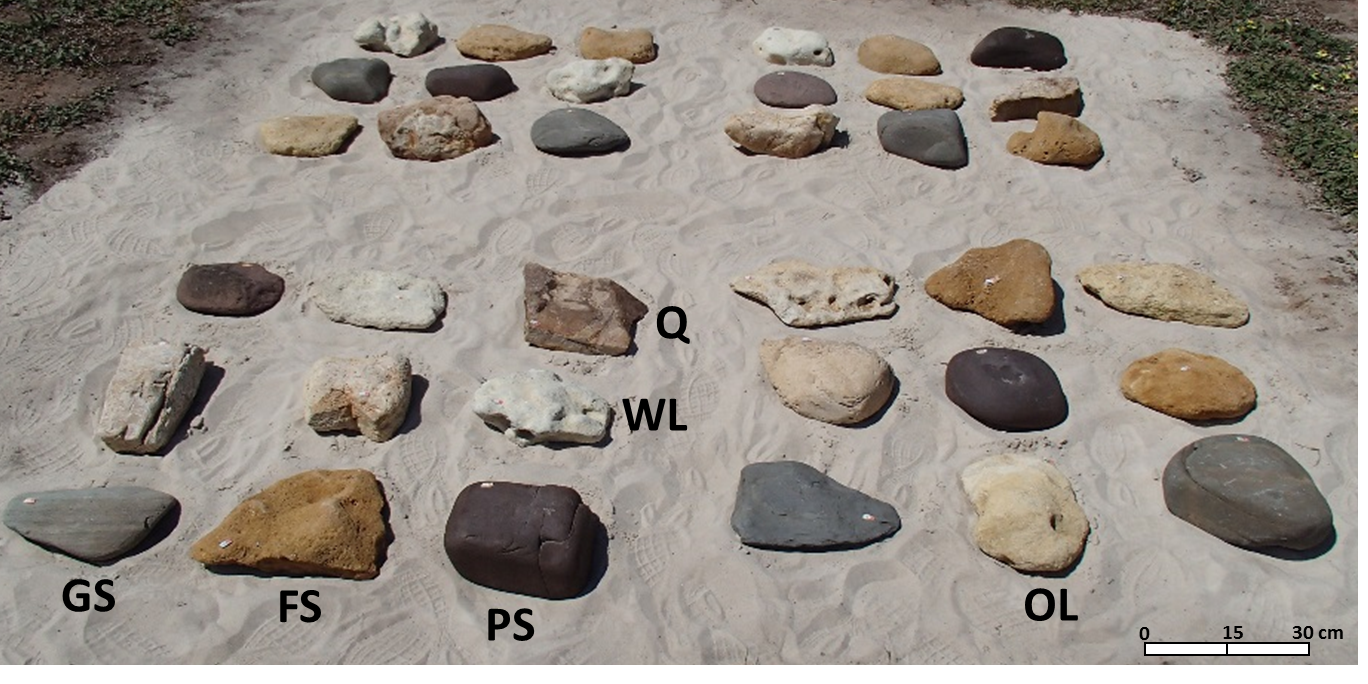


Figure A2: The six rock types in the boulder plot of the common-garden experiment constructed in a paddock at Kangarilla. Q = quartzite; WL = white limestone; GS = grey siltstone; FS = fossiliferous sandstone; PS = purple siltstone; and OL = orange limestone.


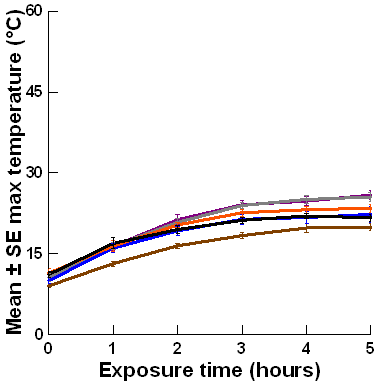

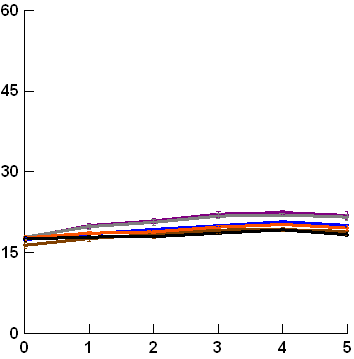

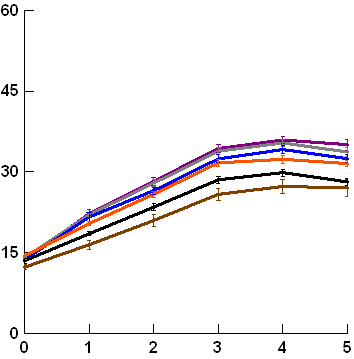

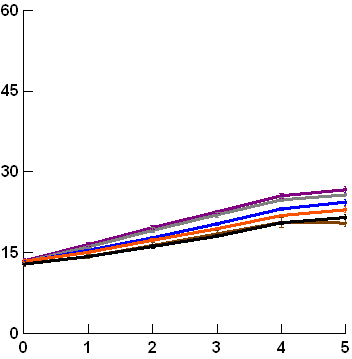

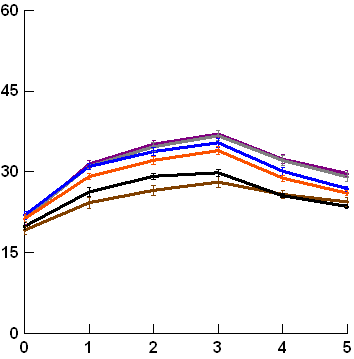

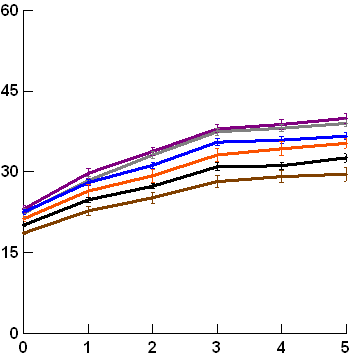

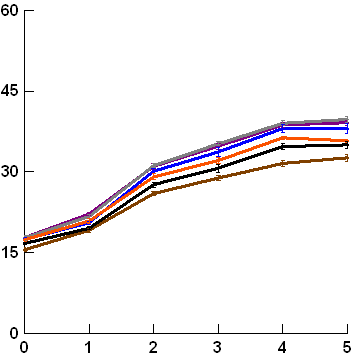

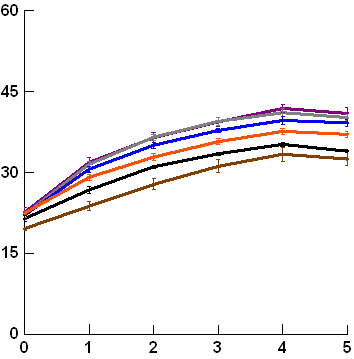


**16/07/2016 (12 °C)**


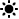

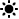

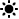

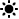

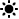

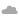

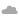

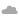


**09/09/2015 (15 °C)**

**10/09/2015 (15 °C)**

**18/09/2015 (17 °C)**

**06/10/2015 (21 °C)**

**17/10/2015 (22 °C)**

**17/04/2017 (23 °C)**

**02/10/2015 (24 °C)**

Figure A3: Mean ± SE maximum upper surface temperature for 6 rock types (*n* = 6 per rock type) over five hours exposure to insolation. The maximum air temperature and weather condition (sunny or cloudy) are specified for each date sampled, with days arranged from coolest to hottest maximum air temperature. Each y-axis extends to encompass the range of raw data.


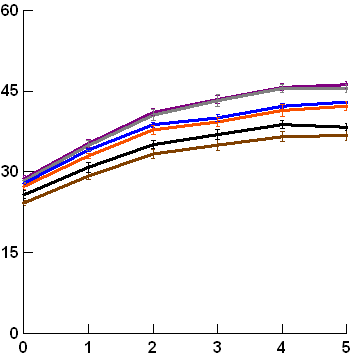

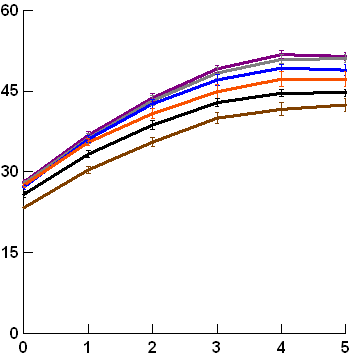

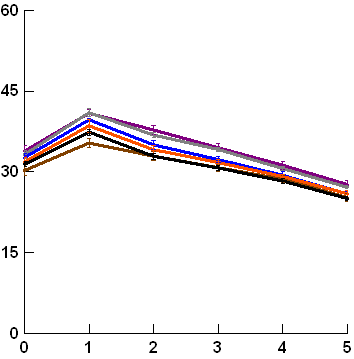

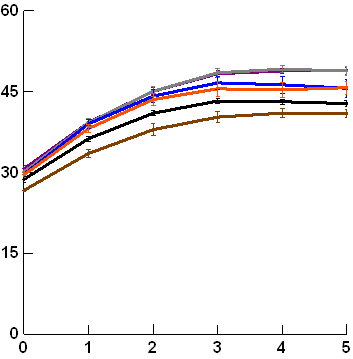

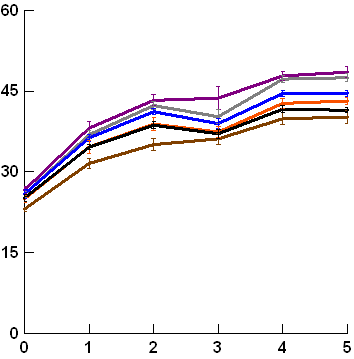

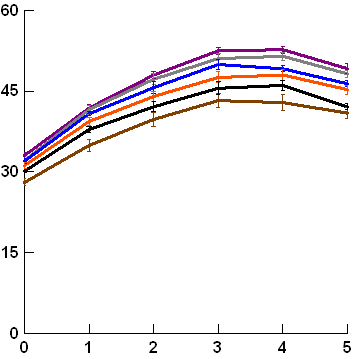

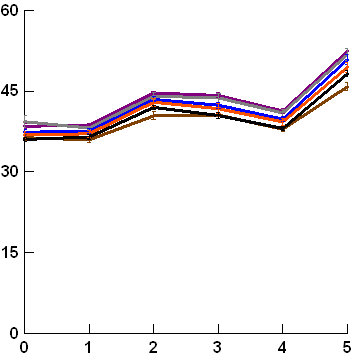

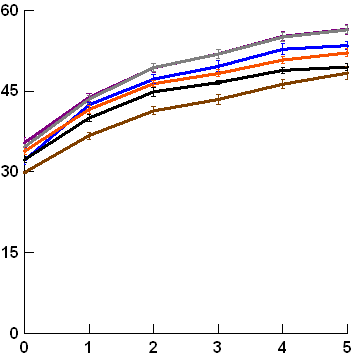

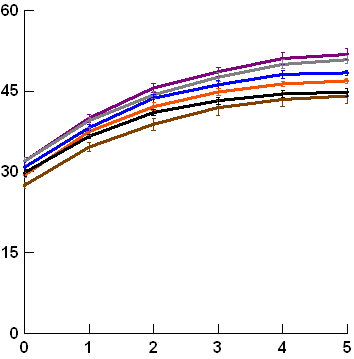


**07/01/2016 (26 °C)**

**07/02/2016 (29 °C)**

**25/11/2015 (30 °C)**

**06/02/2016 (31 °C)**

**09/10/2015 (33 °C)**

**19/11/2015 (34 °C)**

**19/12/2015 (38 °C)**

**08/02/2017 (39 °C)**

**18/11/2015 (40 °C)**


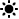

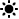

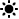

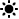

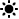

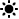

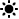

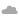

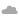


**Purple siltstone**

**Grey siltstone**

**Quartzite**

**Fossiliferous sandstone**

**Orange limestone**

**White limestone**

Figure A3 (continued): Mean ± SE maximum upper surface temperature for 6 rock types (*n* = 6 per rock type) over five hours exposure to insolation. The maximum air temperature and weather condition (sunny or cloudy) are specified for each date sampled, with days arranged from coolest to hottest maximum air temperature. Each y-axis extends to encompass the range of raw data.


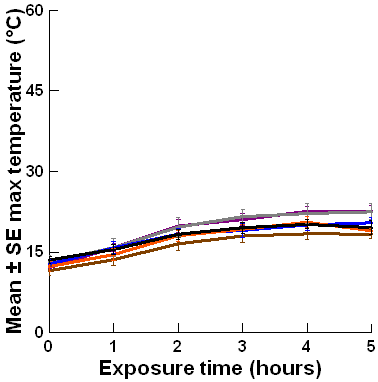

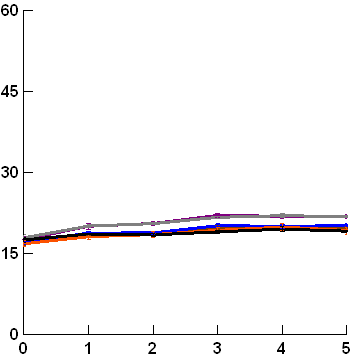

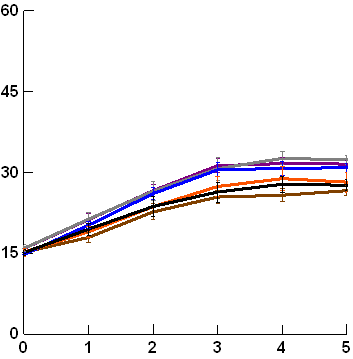

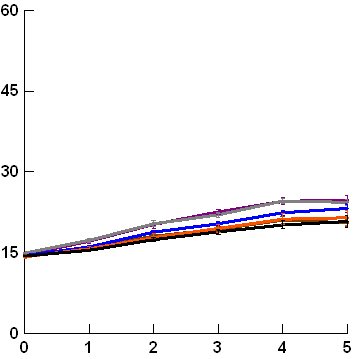

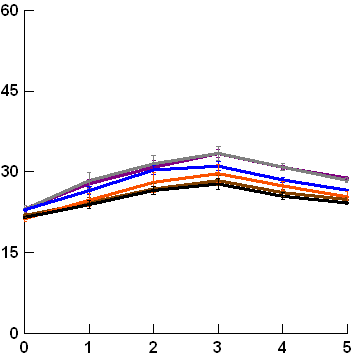

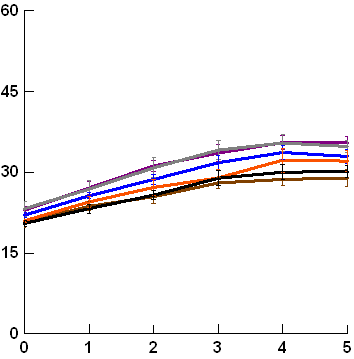

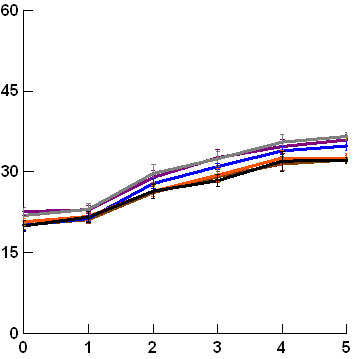

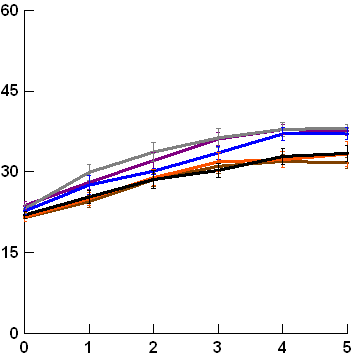


**16/07/2016 (12 °C)**


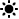

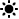

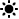

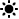

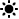

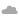

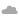

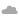


**09/09/2015 (15 °C)**

**10/09/2015 (15 °C)**

**18/09/2015 (17 °C)**

**06/10/2015 (21 °C)**

**17/10/2015 (22 °C)**

**17/04/2017 (23 °C)**

**02/10/2015 (24 °C)**

Figure A4: Mean ± SE maximum lower surface temperature for 6 rock types (*n* = 6 per rock type) over five hours exposure to insolation. The maximum air temperature and weather condition (sunny or cloudy) are specified for each date sampled, with days arranged from coolest to hottest maximum air temperature. Each y-axis extends to encompass the range of raw data.


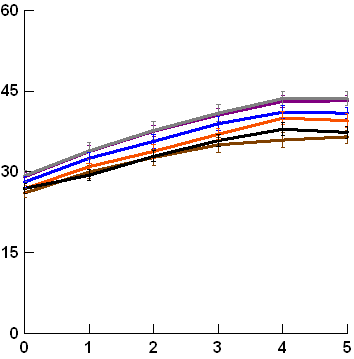

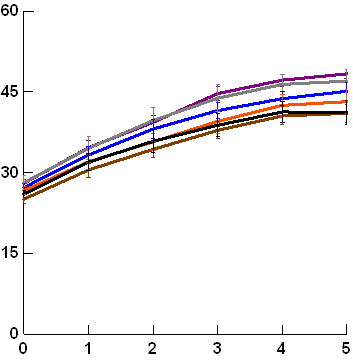

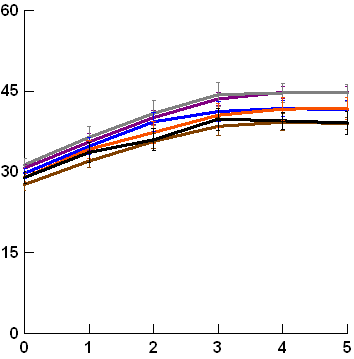

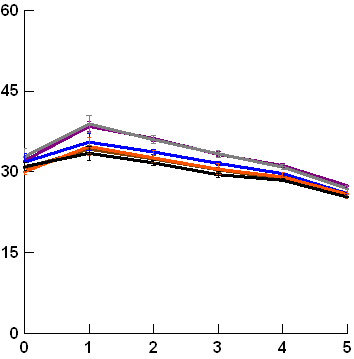

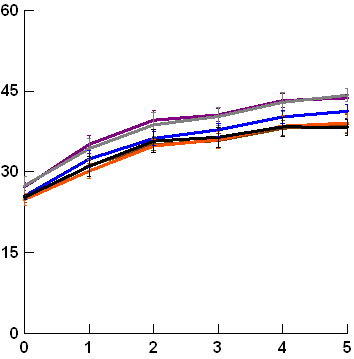

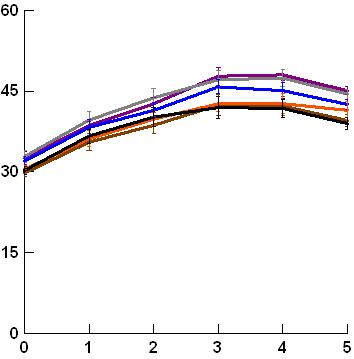

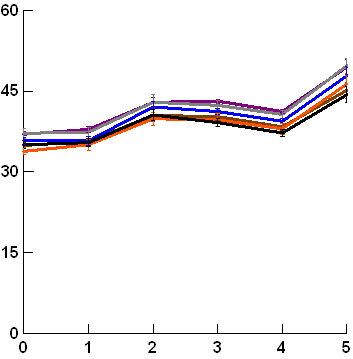

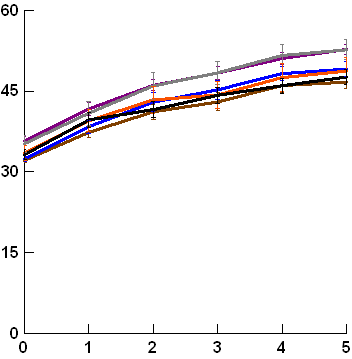

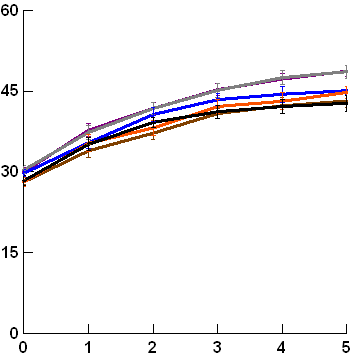


**07/01/2016 (26 °C)**

**07/02/2016 (29 °C)**

**25/11/2015 (30 °C)**

**06/02/2016 (31 °C)**

**09/10/2015 (33 °C)**

**19/11/2015 (34 °C)**

**19/12/2015 (38 °C)**

**08/02/2017 (39 °C)**

**18/11/2015 (40 °C)**


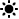

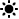

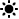

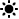

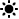

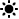

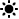

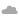

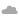


**Purple siltstone**

**Grey siltstone**

**Quartzite**

**Fossiliferous sandstone**

**Orange limestone**

**White limestone**

Figure A4 (continued): Mean ± SE maximum lower surface temperature for 6 rock types (*n* = 6 per rock type) over five hours exposure to insolation. The maximum air temperature and weather condition (sunny or cloudy) are specified for each date sampled, with days arranged from coolest to hottest maximum air temperature. Each y-axis extends to encompass the range of raw data.


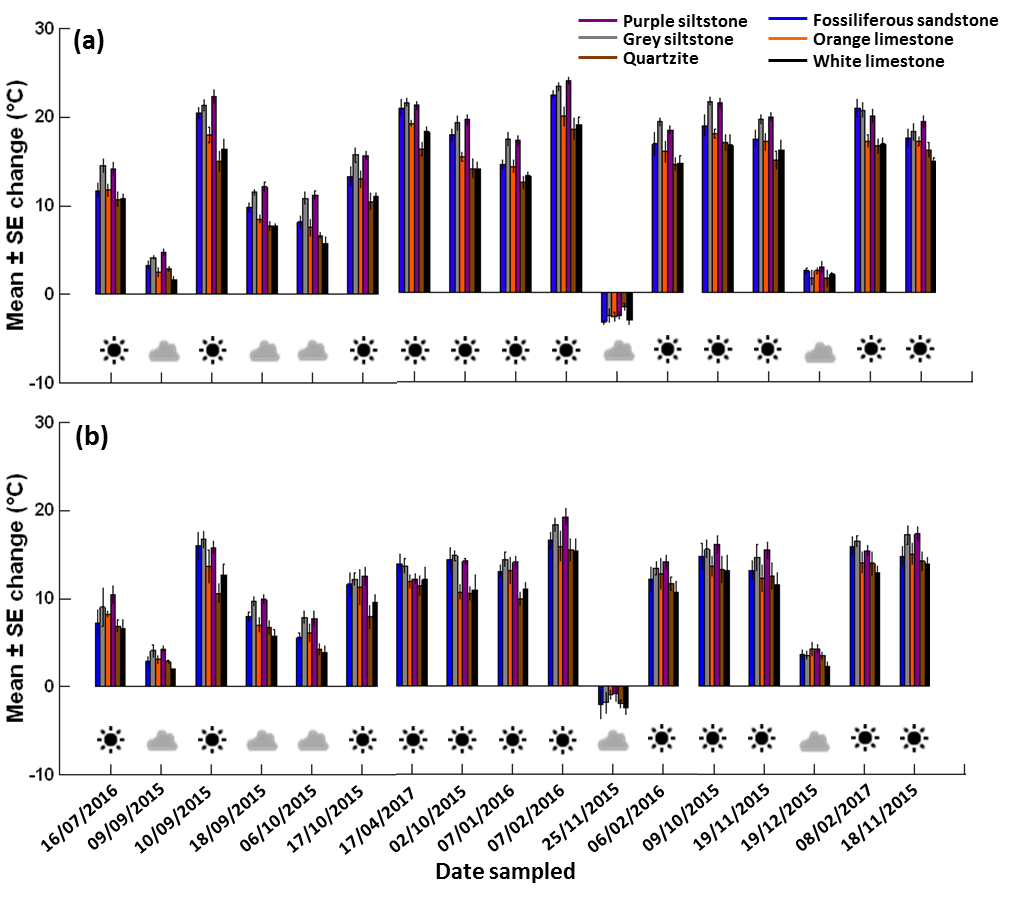


Figure A5: Change over four hours in maxima for each rock type on each day for (a) upper and (b) lower surfaces. Dates on the x-axis are arranged from coolest to hottest maximum air temperatures moving from left to right.


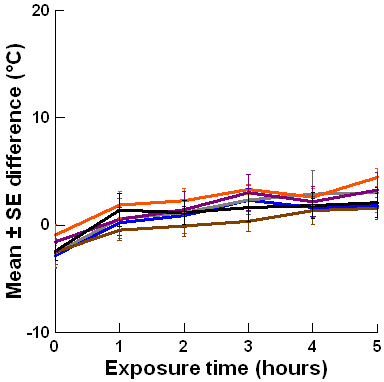

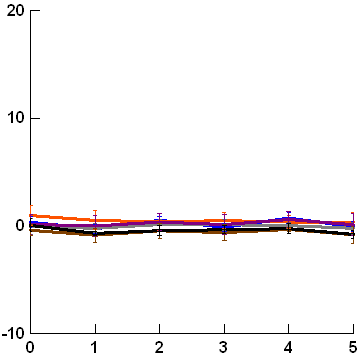

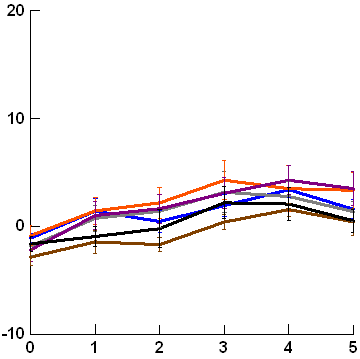

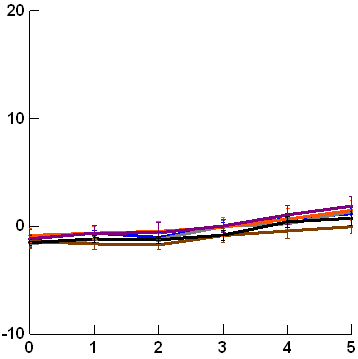

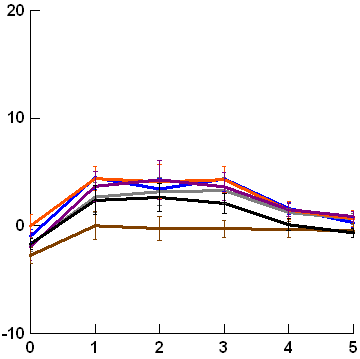

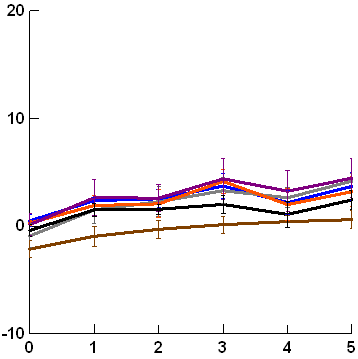

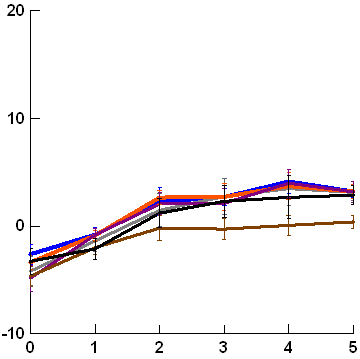

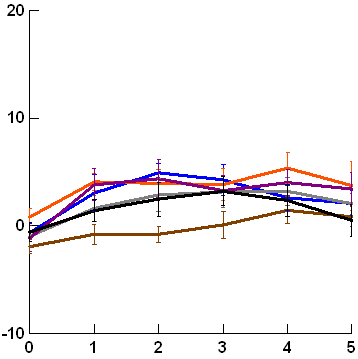


**16/07/2016 (12 °C)**


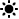

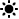

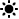

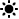

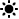

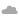

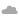

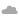


**09/09/2015 (15 °C)**

**10/09/2015 (15 °C)**

**18/09/2015 (17 °C)**

**06/10/2015 (21 °C)**

**17/10/2015 (22 °C)**

**17/04/2017 (23 °C)**

**02/10/2015 (24 °C)**

Figure A6: Mean ± SE maxima difference between boulder upper and lower surfaces for 6 rock types (n = 6 per rock type) over five hours exposure to insolation. The maximum air temperature and weather condition (sunny or cloudy) are specified for each date sampled, with days arranged from coolest to hottest maximum air temperature. Each y-axis extends to encompass the range of raw data.


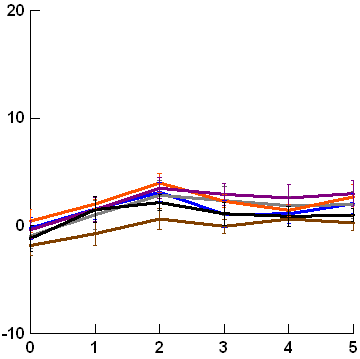

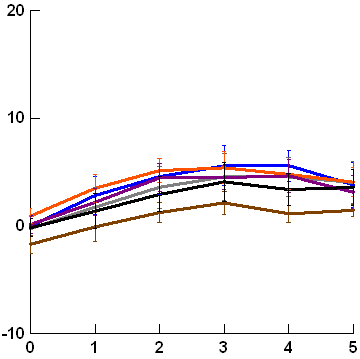

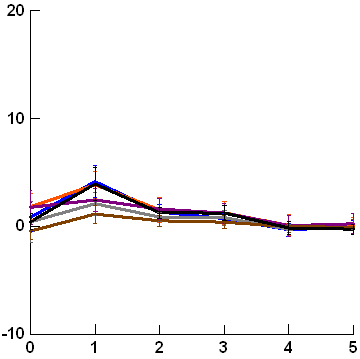

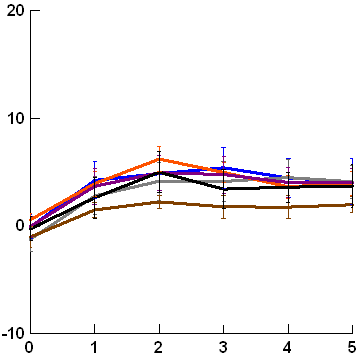

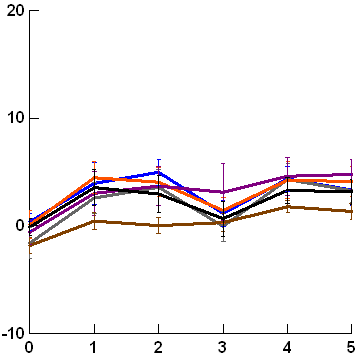

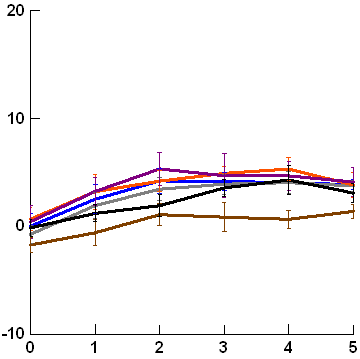

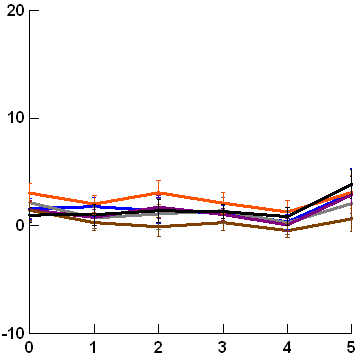

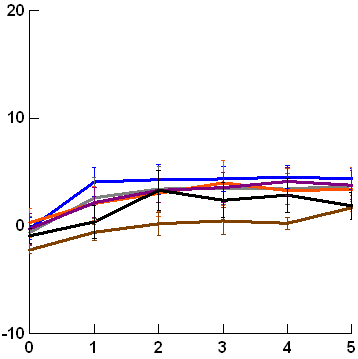

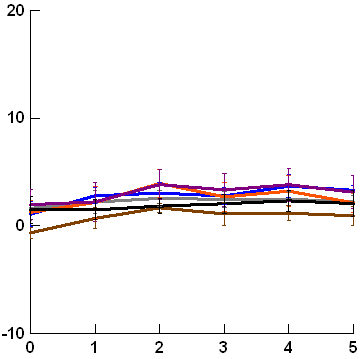


**07/01/2016 (26 °C)**

**07/02/2016 (29 °C)**

**25/11/2015 (30 °C)**

**06/02/2016 (31 °C)**

**09/10/2015 (33 °C)**

**19/11/2015 (34 °C)**

**19/12/2015 (38 °C)**

**08/02/2017 (39 °C)**

**18/11/2015 (40 °C)**


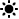

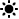

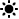

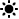


**Purple siltstone**

**Grey siltstone**

**Quartzite**

**Fossiliferous sandstone**

**Orange limestone**

**White limestone**

Figure A6 (continued): Mean ± SE maxima difference between boulder upper and lower surfaces for 6 rock types (n = 6 per rock type) over five hours exposure to insolation. The maximum air temperature and weather condition (sunny or cloudy) are specified for each date sampled, with days arranged from coolest to hottest maximum air temperature. Each y-axis extends to encompass the range of raw data.

**16/07/2016 (12 °C)**

**09/09/2015 (15 °C)**

**10/09/2015 (15 °C)**

**18/09/2015 (17 °C)**

**06/10/2015 (21 °C)**

**17/10/2015 (22 °C)**

**17/04/2017 (23 °C)**

**02/10/2015 (24 °C)**

Figure A7: Mean ± SE upper temperature range for 6 rock types (*n* = 6 per rock type) over five hours exposure to insolation. The maximum air temperature and weather condition (sunny or cloudy) are specified for each date sampled, with days arranged from coolest to hottest maximum air temperature. Each y-axis extends to encompass the range of raw data.

**07/01/2016 (26 °C)**

**07/02/2016 (29 °C)**

**19/11/2015 (34 °C)**

**08/02/2017 (39 °C)**

**06/02/2016 (31 °C)**

**09/10/2015 (33 °C)**

**18/11/2015 (40 °C)**

**25/11/2015 (30 °C)**

**19/12/2015 (38 °C)**

**Purple siltstone**

**Grey siltstone**

**Quartzite**

**Fossiliferous sandstone**

**Orange limestone**

**White limestone**

Figure A7 (continued): Mean ± SE upper temperature range for 6 rock types (*n* = 6 per rock type) over five hours exposure to insolation. The maximum air temperature and weather condition (sunny or cloudy) are specified for each date sampled, with days arranged from coolest to hottest maximum air temperature. Each y-axis extends to encompass the range of raw data.

**16/07/2016 (12 °C)**

**09/09/2015 (15 °C)**

**10/09/2015 (15 °C)**

**18/09/2015 (17 °C)**

**06/10/2015 (21 °C)**

**17/10/2015 (22 °C)**

**17/04/2017 (23 °C)**

**02/10/2015 (24 °C)**

Figure A8: Mean ± SE lower temperature range for 6 rock types (*n* = 6 per rock type) over five hours exposure to insolation. The maximum air temperature and weather condition (sunny or cloudy) are specified for each date sampled, with days arranged from coolest to hottest maximum air temperature. Each y-axis extends to encompass the range of raw data.

**07/01/2016 (26 °C)**

**07/02/2016 (29 °C)**

**19/11/2015 (34 °C)**

**08/02/2017 (39 °C)**

**06/02/2016 (31 °C)**

**09/10/2015 (33 °C)**

**18/11/2015 (40 °C)**

**25/11/2015 (30 °C)**

**19/12/2015 (38 °C)**

**Purple siltstone**

**Grey siltstone**

**Quartzite**

**Fossiliferous sandstone**

**Orange limestone**

**White limestone**

Figure A8 (continued): Mean ± SE lower temperature range for 6 rock types (*n* = 6 per rock type) over five hours exposure to insolation. The maximum air temperature and weather condition (sunny or cloudy) are specified for each date sampled, with days arranged from coolest to hottest maximum air temperature. Each y-axis extends to encompass the range of raw data.

Figure A9: Change over four hours in temperature range for each rock type on each day for (a) upper and (b) lower surfaces. Dates on the x-axis are arranged from coolest to hottest maximum air temperatures moving from left to right.

**16/07/2016 (12 °C)**

**09/09/2015 (15 °C)**

**10/09/2015 (15 °C)**

**18/09/2015 (17 °C)**

**06/10/2015 (21 °C)**

**17/10/2015 (22 °C)**

**17/04/2017 (23 °C)**

**02/10/2015 (24 °C)**

Figure A10: Mean ± SE temperature range difference between boulder upper and lower surfaces for 6 rock types (*n* = 6 per rock type) over five hours exposure to insolation. The maximum air temperature and weather condition (sunny or cloudy) are specified for each date sampled, with days arranged from coolest to hottest maximum air temperature. Each y-axis extends to encompass the range of raw data.

**07/01/2016 (26 °C)**

**07/02/2016 (29 °C)**

**19/11/2015 (34 °C)**

**08/02/2017 (39 °C)**

**06/02/2016 (31 °C)**

**09/10/2015 (33 °C)**

**18/11/2015 (40 °C)**

**25/11/2015 (30 °C)**

**19/12/2015 (38 °C)**

**Purple siltstone**

**Grey siltstone**

**Quartzite**

**Fossiliferous sandstone**

**Orange limestone**

**White limestone**

Figure A10 (continued): Mean ± SE temperature range difference between boulder upper and lower surfaces for 6 rock types (*n* = 6 per rock type) over five hours exposure to insolation. The maximum air temperature and weather condition (sunny or cloudy) are specified for each date sampled, with days arranged from coolest to hottest maximum air temperature. Each y-axis extends to encompass the range of raw data.
